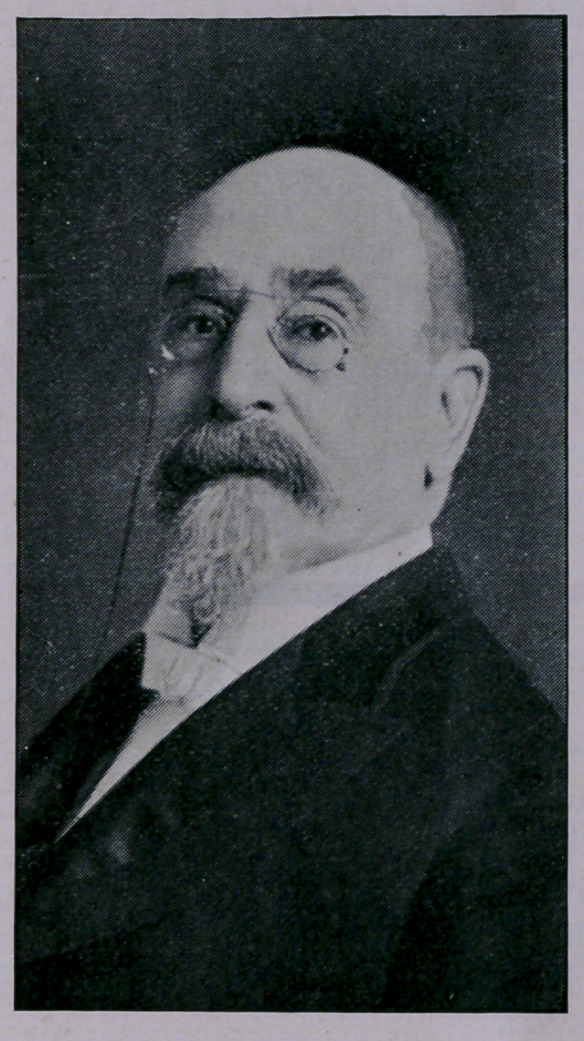# Dr. Arthur S. Wolff, Dean of the Texas Quarantine Service

**Published:** 1904-11

**Authors:** F. E. Daniel


### DR. ARTHUR S. WOLFF, DEAN OF THE TEXAS QUARANTINE SERVICE.

The venerable and beloved Dr. A. S. Wolff is dead. He died at his home at Brownsville, Texas, October 30th (ult.), at the age of 85 years, 8 months and 18 days. He was born at Lyons, France, in 1819. His father, Dr. Simeon Wolff, was a noted physician of Paris. Dr. Arthur Wolff was educated at the famous Montpelier School in Paris—receiving two medals for proficiency, and the degree A. B. He then took the prescribed nine years' course at the France Academy of Medicine—receiving the degree M. D. P. Entered the French army as surgeon. As such he served with the Third Zouaves in Algeria in 1846. For services in this campaign he was decorated with the Cross of the Legion of Honor. Resigning, he took a course at the University of Leyden, and received the degree M. D. from that famous institution. He then engaged in private practice in England, and immigrated to America in 1859, settling in New York.

On the breaking out of the Civil War he was appointed Surgeon to Fifty-fifth N. Y. Volunteers; whence he was transferred to Lincoln Hospital, Washington City. After the battle of Gettysburg, Dr. Wolff again entered the field, serving with Fifth Army Corps under General Sykes. Returning to Washington, he served in Carver Hospital till end of the war. Was Surgeon to Clinton Prison, N. Y., in 1867. Went to Texas in 1875, settling in Brownsville, where he continued to reside till date of his death. In 1877 he was appointed by Governor Hubbard Quarantine Officer at that port, which position he continuously held and filled under every Texas Governor and State Health Officer to date of his death, and died, after more than a quarter century's service, in the active discharge of its duties.

His territory was very large, his duties very arduous, and often laborious, involving journeys of hundreds of miles by private conveyance overland, but he always responded to the call of duty cheerfully, and discharged his official duties efficiently and fearlessly.

There could be no higher evidence of Dr. Wolff's great ability and worth; of his fidelity and his zeal in the cause of the public health than this unbroken record of appointment and reappointment to the responsible position by Governors Hubbard, Roberts, Ireland, Ross, Hogg, Culberson, Sayers and Lanham.

Dr. Wolff was married in London in 1850 to Miss Sarah Ansell. Three of his children were born in London—Carrie, Arthur and Blanch. His son, Dr. Arthur Wolff, Jr., is Bacteriologist of the Connecticut Board of Health and is in general practice at Hartford. Dr. Harry K. Loew, of Brownsville, graduate of Texas Medical College is his grandson. His wife and children and several grandchildren survive him. His daughters are Mrs. S. J. Zanders, of Brooklyn, N. Y., Mrs. Blanch R. Leow, Brownsville, and Mrs. Bernard L. Cain, Brownsville.

Dr. Wolff is a great loss to the profession and to the State. I knew him well and loved him for his worth. He was a princely man, a genial and delightful companion, and in every relation of life was altogether admirable.

F. E. Daniel.

## Figures and Tables

**Figure f1:**